# PEITC-mediated inhibition of mRNA translation is associated with both inhibition of mTORC1 and increased eIF2α phosphorylation in established cell lines and primary human leukemia cells

**DOI:** 10.18632/oncotarget.11655

**Published:** 2016-08-27

**Authors:** Alison Yeomans, Elizabeth Lemm, Sarah Wilmore, Breeze E. Cavell, Beatriz Valle-Argos, Sergey Krysov, Marina Sanchez Hidalgo, Elodie Leonard, Anne E. Willis, Francesco Forconi, Freda K. Stevenson, Andrew J. Steele, Mark J. Coldwell, Graham Packham

**Affiliations:** ^1^ Cancer Research UK Centre, Faculty of Medicine, University of Southampton, Southampton, UK; ^2^ MRC Toxicology Unit, Leicester, UK; ^3^ Haematology Oncology Group, Cancer Sciences Unit, Cancer Research UK Centre, University of Southampton, Faculty of Medicine, Southampton, UK; ^4^ Department of Haematology, University Hospital Southampton NHS Trust, Southampton, UK; ^5^ Centre for Biological Sciences, Faculty of Natural and Environmental Sciences, University of Southampton, Southampton, UK; ^6^ Current Address: Public Health England, Porton Down, Salisbury, UK; ^7^ Current Address: Department of Pharmacology, Faculty of Pharmacy, University of Seville, Seville, Spain; ^8^ Current Address: XPE Pharma and Science, Wavre, Belgium; ^9^ Current Address: Bart's Cancer Institute, Queen Mary University of London, London, UK

**Keywords:** phenethylisothiocyanate, mRNA translation, eIF2α, mTORC1, MYC

## Abstract

Increased mRNA translation drives carcinogenesis and is an attractive target for the development of new anti-cancer drugs. In this work, we investigated effects of phenethylisothiocyanate (PEITC), a phytochemical with chemopreventive and anti-cancer activity, on mRNA translation. PEITC rapidly inhibited global mRNA translation in human breast cancer-derived MCF7 cells and mouse embryonic fibroblasts (MEFs). In addition to the known inhibitory effects of PEITC on mTORC1 activity, we demonstrate that PEITC increased eIF2α phosphorylation. PEITC also increased formation of stress granules which are typically associated with eIF2α phosphorylation and accumulation of translationally stalled mRNAs. Analysis of genetically modified MEFs demonstrated that optimal inhibition of global mRNA translation by PEITC was dependent on eIF2α phosphorylation, but not mTORC1 inhibition. We extended this study into primary leukemic B cells derived from patients with chronic lymphocytic leukaemia (CLL). CLL cells were stimulated *in vitro* with anti-IgM to mimic binding of antigen, a major driver of this leukemia. In CLL cells, PEITC increased eIF2α phosphorylation, inhibited anti-IgM-induced mTORC1 activation and decreased both basal and anti-IgM-induced global mRNA translation. PEITC also inhibited transcription and translation of *MYC* mRNA and accumulation of the MYC oncoprotein, in anti-IgM-stimulated cells. Moreover, treatment of CLL cells with PEITC and the BTK kinase inhibitor ibrutinib decreased anti-IgM-induced translation and induced cell death to a greater extent than either agent alone. Therefore, PEITC can inhibit both global and mRNA specific translation (including MYC) via effects on multiple regulatory pathways. Inhibition of mRNA translation may contribute to the chemopreventive and anti-cancer effects of PEITC.

## INTRODUCTION

Phenethyl isothiocyanate (PEITC) is a naturally occurring phytochemical that has received extensive interest for its anti-cancer and chemopreventive activities. [1] PEITC induces cell cycle arrest and apoptosis, and inhibits metastasis, proliferation and angiogenesis in various malignant cell types and, therefore, has inhibitory effects on multiple cancer hallmarks. The compound is currently undergoing clinical evaluation for chemopreventive activity, including a phase II trial in lung cancer.

Recent findings have indicated that PEITC inhibits mRNA translation [2–4]. mRNA translation has a high energy requirement and is subject to tight regulation, especially at the level of initiation. In fact, recent analyses have suggested that regulation of mRNA translation may play a role as great as transcription in controlling protein expression [5]. Translation initiation is mediated by eukaryotic initiation factors (eIF) that recruit the two subunits of the ribosome to the correct mRNA start codon and ensure delivery of the initiator methionine-loaded tRNA [6]. mRNA translation is classically initiated in a 5′-cap-dependent manner, where the 5′-cap of mRNA is recognized by the eIF4F complex comprising the 5′-cap-binding protein, eIF4E, a helicase, eIF4A, and the scaffold protein, eIF4G. Additional eIFs are then recruited to modulate translation initiation rate and stringency of start codon selection. For example, eIF2B acts as a guanine nucleotide exchange factor for eIF2 (a trimer comprising α, β and γ subunits) and catalyzes the exchange of GDP to GTP that is required to form an active ternary complex after each round of translation initiation.

Modulation of eIF4E and eIF2 are pivotal for controlling mRNA translation [7]. eIF4E activity is regulated by the mTORC1 kinase complex via effects on eIF4E-binding proteins (4E-BPs) that sequester and inactivate eIF4E [8] and through direct phosphorylation of eIF4E by MAP kinase-integrating kinases (MNKs). [9] mTORC1-mediated 4E-BP phosphorylation triggers eIF4E release allowing binding to the mRNA 5′-cap and interaction with eIF4G. mTORC1 also regulates translation via p70S6K phosphorylation, thereby modulating ribosomal protein S6 activity [10, 11]. Initiation is also regulated by Ser51 phosphorylation of eIF2α. When phosphorylated at this site, eIF2α acts as a competitive inhibitor of eIF2B, thereby preventing ternary complex recycling and inhibiting translation [12]. eIF2α phosphorylation is typically induced as part of a stress response. For example, endoplasmic reticulum (ER) stress activates the eIF2α Ser51-specific kinase PERK leading to suppression of mRNA translation [12].

Dysregulation of mRNA translation plays a major role in cancer, acting to drive cell accumulation via effects on both global protein and specific proto-oncogenes which often have high requirements for translation [13]. Translationally regulated oncoproteins include MYC, a central regulator of cell growth, and HIF1A, which mediates pro-angiogenic responses in hypoxic conditions. Dysregulated mRNA translation in cancer cells is often associated with high levels of eIF4E, and eIF4E overexpression is sufficient to drive lymphomagenesis [14]. mTORC1 activation, associated with loss of negative regulatory tumor suppressor proteins (such as NF1 or PTEN), or inappropriate growth factor signaling, is also common in cancers. Overexpression of oncoproteins such as MYC can also lead to inappropriate mRNA translation [15] and eIF4E accelerates development of MYC-driven lymphoma [16]. Thus, MYC is intimately linked to mRNA translation since it is both a target for, and a major regulator of, mRNA translation. Inhibition of mRNA translation is, therefore, an exciting new approach to cancer therapy [17]. In fact, a recent study demonstrated the effectiveness of chemical inhibition of mRNA translation as a strategy to counter MYC-driven tumorigenesis *in vivo*, whereas inhibition of signaling upstream of MYC was circumvented by complex cross-talk mechanisms [18].

Despite the importance of dysregulated protein synthesis for cancer, few studies have investigated effects of PEITC on these pathways. Hu et al. demonstrated that PEITC increased eIF4E expression and decreased 4E-BP1 phosphorylation in PC-3 prostate cancer cells, and inhibited 5′-cap-dependent mRNA translation measured using artificial reporter assays [2]. More recently, we demonstrated that PEITC decreased HIF1α protein expression via inhibition of *HIF1A* mRNA translation in MCF7 breast cancer cells [3, 4]. This inhibitory effect appeared to be driven via inhibition of mTORC1 [4] which is required for optimal *HIF1A* mRNA translation [19, 20].

Studies of translational regulation have almost exclusively used established cell lines. Although clearly of great value, it is possible that regulatory pathways are altered in these settings since long-term culture will select for more metabolically active cell variants. Therefore, analysis of mRNA translation in primary cancer cells is an important goal. Chronic lymphocytic leukemia (CLL) provides a powerful model system for the detailed molecular analysis of primary cancer cells. It is the most common B-cell malignancy [21] and provides access to large numbers of monoclonal malignant B cells from the blood of patients.

Antigenic stimulation of the cell surface B-cell receptor (BCR) is a major driver of malignant cell accumulation *in vivo* in CLL. BCR signaling responsiveness varies between individual samples and retained signaling capacity is associated with a poor outcome. Moreover, inhibitors of BCR-associated signaling kinases (such as the BTK inhibitor ibrutinib) are revolutionising therapy for B-cell malignancies [22]. Antigenic stimulation can be mimicked using agonistic anti-IgM antibodies and we showed previously that anti-IgM increased MYC expression in CLL cells *in vitro* and that MYC was expressed in lymph nodes from CLL patients, the site of antigen engagement *in vivo* [23]. More recently we demonstrated that anti-IgM increased both global mRNA translation and translation of *MYC* mRNA in primary CLL cells *in vitro* [24]. These responses were partially inhibited by ibrutinib. Therefore, CLL is a well validated model to study translational control in primary malignant cells.

In this work, we investigated effects of PEITC on mRNA translation. We show that, in addition to inhibition of mTORC1, PEITC triggers rapid phosphorylation of eIF2α and that eIF2α phosphorylation is required for optimal PEITC-mediated translational inhibition in mouse embryo fibroblasts (MEFs). PEITC also inhibited both basal and anti-IgM-induced mRNA translation in primary CLL cells (including translation of the *MYC* mRNA) and this was associated with both mTORC1 inhibition and increased eIF2α phosphorylation.

## RESULTS

### PEITC inhibits mRNA translation in MCF7 cells in a dose and time dependent manner

**Figure 1:**
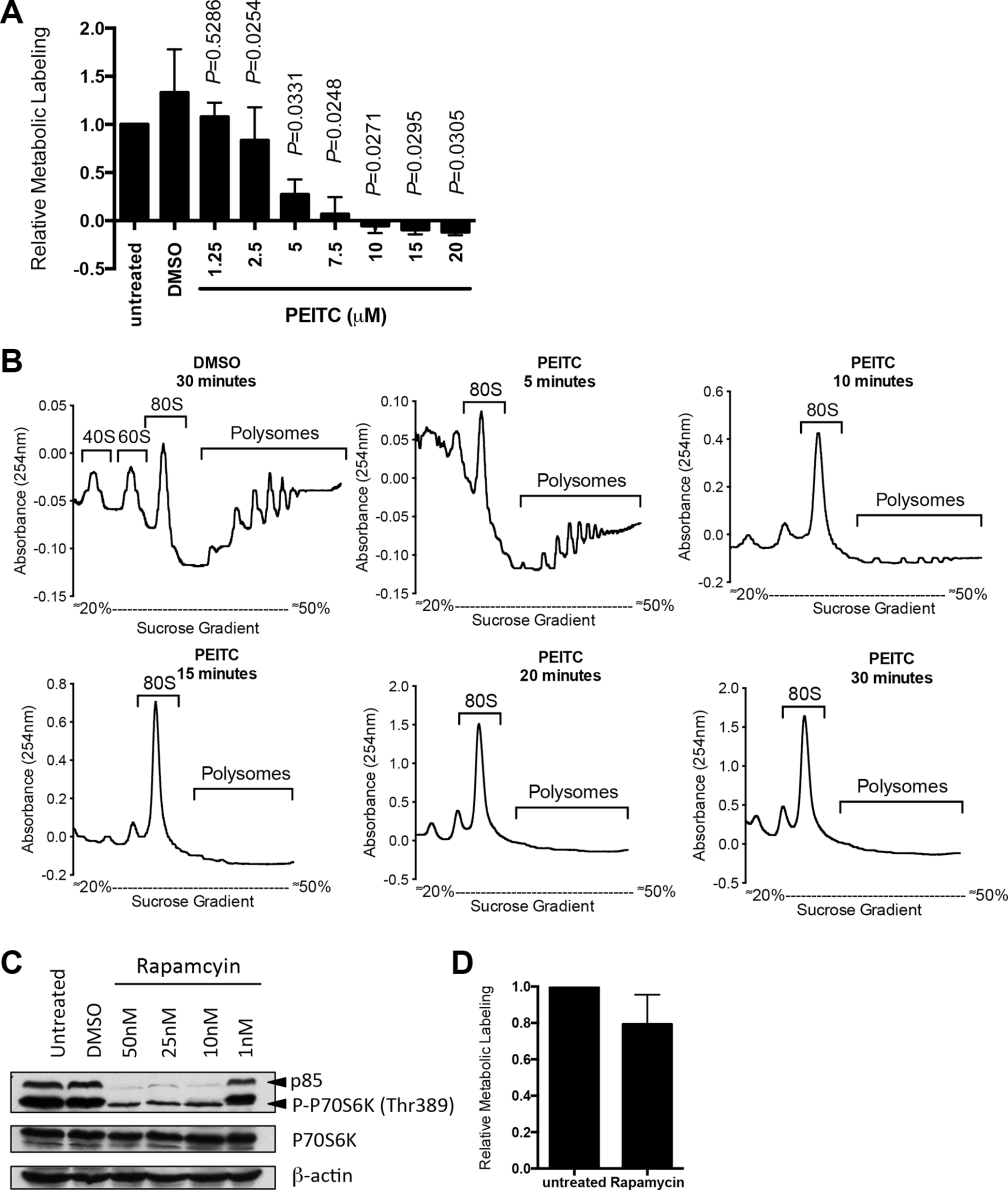
PEITC inhibits global mRNA translation in MCF7 cells (**A**) MCF7 cells were incubated with the indicated concentrations of PEITC, DMSO (solvent control), or were left untreated as a control. After one hour, mRNA translation was quantified using metabolic labeling. Graph shows means (± SEM) derived from three independent experiments, each performed in duplicate, with values for untreated cells set to 1.0. Statistical significance of differences between PEITC and DMSO treated cells is shown (Student's *t*-test). (**B**) MCF7 cells were incubated with PEITC (20 μM) or DMSO as a control. After the indicated times, polysome profiling was performed. The position of 80S and polysomes are indicated. Data are representative of three separate experiments. (**C**) MCF7 cells were treated with the indicated concentrations of rapamycin, DMSO, or were left untreated as a control. After three hours, expression of total and phosphorylated p70S6K and β-actin (loading control) was analyzed using immunoblotting. Experiment shown is representative of three independent experiments. (**D**) MCF7 cells were incubated with rapamycin (25 nM) or left untreated as a control. After one hour, mRNA translation was quantified using metabolic labeling. Graph shows means (± SEM) derived from duplicate determinations, with values for untreated cells set to 1.0.

We first investigated effects of PEITC on global mRNA translation in human breast cancer-derived MCF7 cells using metabolic labeling and polysome profiling. PEITC was used at concentrations up to 20 μM, based on previous published studies [4, 25]. PEITC profoundly inhibited metabolic labeling (Figure 1A). Inhibitory effects were dose-dependent with half-maximal response at between 2.5 μM and 5 μM. When evaluated using polysome profiling, PEITC (20 μM) completely blocked formation of polysomes (actively translated mRNA associated with multiple ribosomes) with concurrent accumulation of mRNA in the 80S monosome peak (Figure 1B and Supplementary Figure S1A). Inhibition of polysome formation was essentially complete at 10 minutes post-treatment. Therefore, PEITC triggers a profound and rapid inhibition of global mRNA translation in MCF7 cells.

We previously showed that PEITC inhibited mTORC1 activity [4]. Although this would be expected to reduce mRNA translation, mTORC1 inhibition generally has modest inhibitory effects on global mRNA translation since it predominantly affects a subset of mRNAs whose 5'-untranslated region (5'-UTR) contains highly structured 5′-terminal oligopyrimidine motifs, including *HIF1A* [26]. To determine directly whether mTORC1 inhibition could account for the profound inhibition of mRNA translation induced by PEITC, we investigated effects of the mTORC1 inhibitor, rapamycin. Analysis of phosphorylation of the mTORC1 substrate p70S6K confirmed effectiveness of the inhibitor at concentrations down to 1 nM (Figure 1C). However, even when tested at 25 nM, rapamycin only modestly reduced mRNA translation in MCF7 cells measured using metabolic labeling (Figure 1D) or polysome profiling (Supplementary Figure S1B). Therefore, PEITC-mediated translational inhibition must involve other targets in addition to mTORC1.

### PEITC induces eIF2α phosphorylation and induces stress granule formation

**Figure 2:**
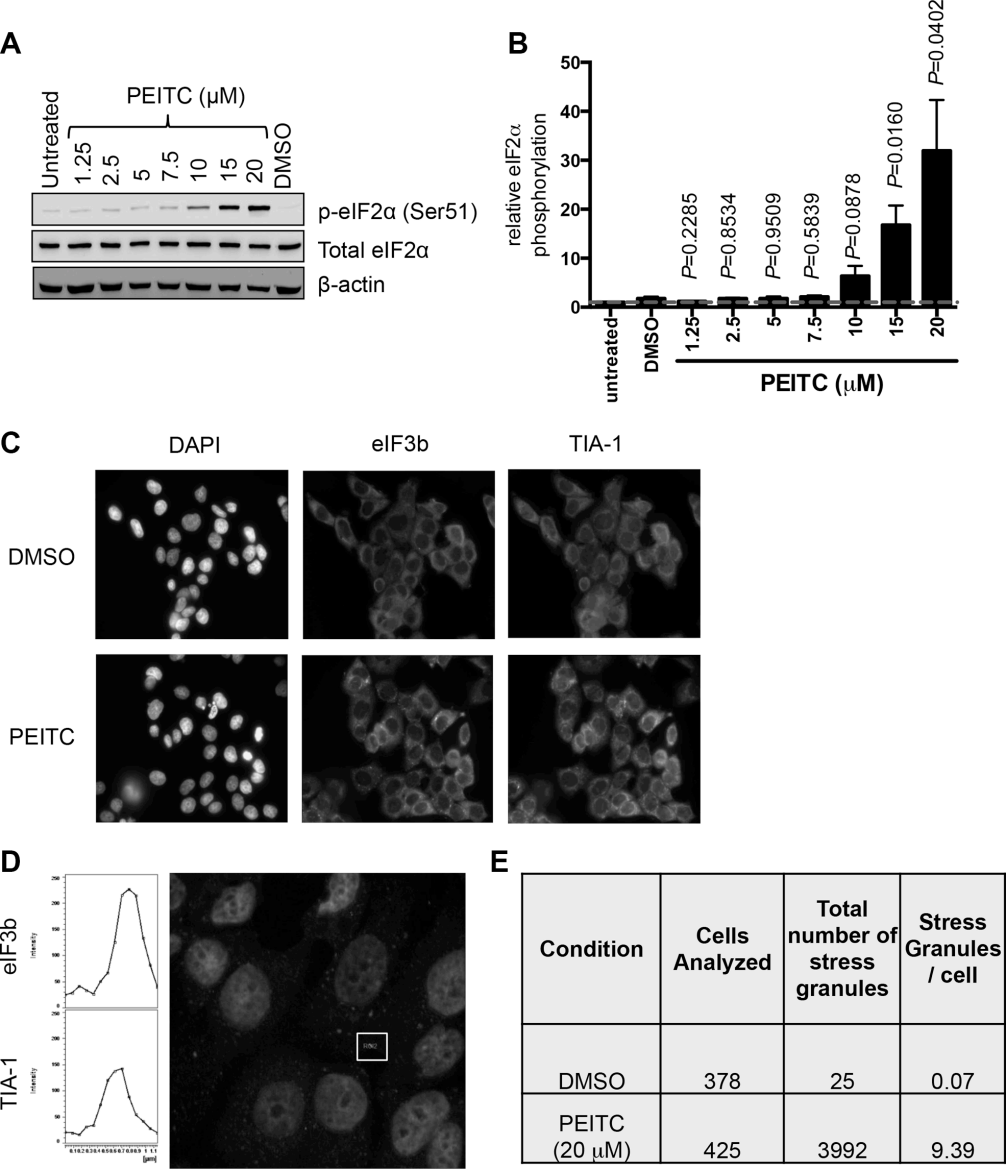
PEITC induces eIF2α Ser51 phosphorylation and stress granule formation (**A**, **B**) MCF7 cells were treated with the indicated concentrations of PEITC, DMSO, or were left untreated as a control. After three hours, total and phosphorylated eIF2α and β-actin were analyzed by immunoblotting. (A) Representative experiment and (B) quantitation, derived from three independent experiments each performed in duplicate. Graph shows means (± SEM) with values for untreated cells set to 1.0. Statistical significance of differences between PEITC and DMSO treated cells is shown (Student's *t*-test). (**C**–**E**) MCF7 cells were treated with PEITC (20 μM) or DMSO for 30 minutes. (C) Immunofluorescence images for DAPI (nuclear stain), eIF3 and TIA-1 staining. Images are representative of three independent experiments. (D) Confocal microscopy. The fluorescent images show merged results for eIF3 and TIA-1 with overlapping signals shown in yellow (Figure S3). Left hand panels show cross-sectional intensities for eIF3 and TIA-1 staining for one stress granule (indicated). (E) Quantitation of eIF3/TIA-1 positive stress granules. Results obtained from analysis of four fields each from three independent experiments. A color version of C and D is presented as Figure S3.

We next investigated effects of PEITC on phosphorylation of eIF2α, a second major regulatory arm for mRNA translation. Immunoblot analysis demonstrated that PEITC induced strong eIF2α phosphorylation in MCF7 cells (Figure 2A, 2B). Similar to inhibition of mRNA translation, induction of eIF2α phosphorylation was concentration dependent (Figure 2A, 2B) and rapid (Supplementary Figure S2). Therefore, in addition to mTORC1 inhibition, PEITC also promotes rapid eIF2α phosphorylation.

To begin to probe the functional significance of PEITC-induced eIF2α phosphorylation, we investigated the potential formation of stress granules, which is tightly linked to increased eIF2α phosphorylation [27]. Stress granules are cytoplasmic aggregates of stalled pre-initiation complexes, containing translationally silenced mRNA, the 40S ribosomal unit and some eIFs. Following treatment with PEITC (20 μM), immunofluorescent imaging demonstrated co-localisation of the two specific stress granule markers, eIF3b and TIA-1, [28] within cytoplasmic complexes (Figure 2C, 2D and Supplementary Figure S3). Stress granules were infrequent in control cells (less than 1 in 10 cells) but abundant following PEITC treatment (~10 granules per cell; Figure 2E). The number of stress granules induced by 10 μM PEITC was similar to that induced by thapsigargin (40 μM), a known inducer of eIF2α phosphorylation and stress granule formation (data not shown).

### PEITC-induced eIF2α phosphorylation is required for optimal inhibition of global mRNA translation

**Figure 3:**
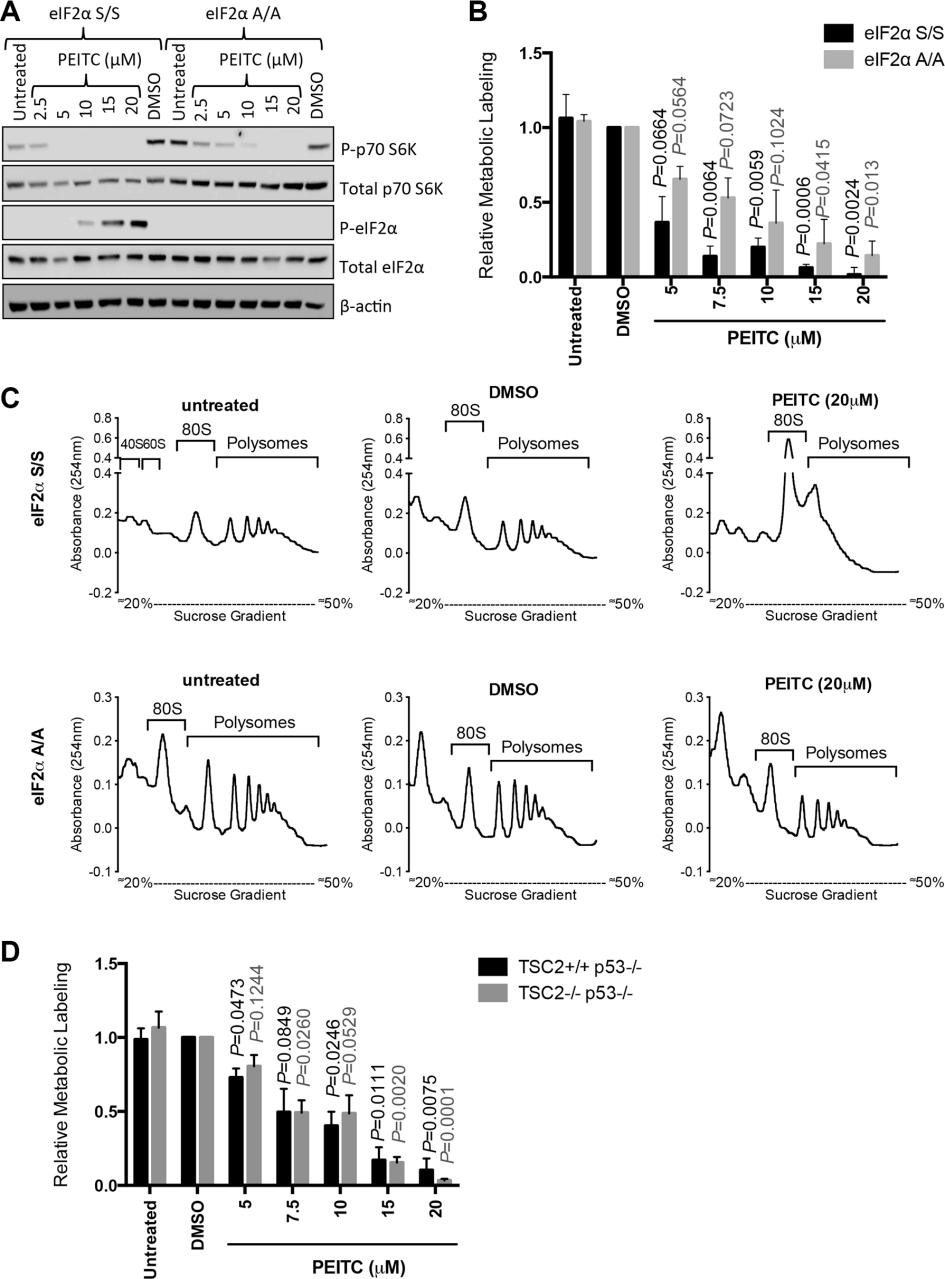
eIF2α phosphorylation is required for optimal PEITC-induced inhibition of mRNA translation (**A**–**C**) eIF2α S/S and eIF2α A/A MEFs were treated with indicated concentrations of PEITC, DMSO, or were left untreated as a control. (A) After three hours, total and phosphorylated p70S6K and eIF2α, and β-actin were analyzed by immunoblotting. (B) After one hour, mRNA translation was quantified using metabolic labeling incubation for a further two hours giving a total PEITC treatment time of three hours. Graph shows means (± SEM) derived from three independent experiments, each performed in duplicate, with values for DMSO treated cells set to 1.0. Statistical significance of differences between PEITC and DMSO treated cells for each line is shown (Student's *t*-test). (C) After one hour, mRNA translation was analyzed using by polysome profiling. Experiments shown are representative of three independent experiments. (**D**) As for (B), but using TSC2^+/+^p53^−/−^ and TSC2^−/−^p53^−/−^ MEFs.

To investigate directly the relevance of eIF2α phosphorylation for PEITC-induced translational inhibition, we analyzed responses in MEFs expressing either wild-type eIF2α (S/S) or mutant eIF2α (with alanine substitution of Ser51) which cannot undergo Ser51 phosphorylation (A/A) [29]. Similar to MCF7 cells, PEITC induced eIF2α phosphorylation in S/S cells, but (as expected) not in A/A cells (Figure 3A). By contrast, PEITC-induced inhibition of mTORC1, measured by p70S6K phosphorylation, was similar in S/S cells and A/A cells.

We used metabolic labeling to quantify mRNA translation in eIF2α S/S and A/A cells. PEITC inhibited metabolic labeling in both cell types, but A/A cells were clearly less sensitive compared to S/S cells (Figure 3B). Similar results were obtained using polysome profiling where PEITC ablated polysome formation in S/S cells, but had partial effects in A/A cells (Figure 3C). Therefore, eIF2α phosphorylation is required for optimal PEITC-induced inhibition of mRNA translation.

We also used metabolic labeling to directly investigate the role of mTORC1 in PEITC-mediated inhibition of global mRNA translation. We compared inhibitory effects of PEITC in wild-type MEFs, and MEFS lacking TSC2, an upstream negative regulator of mTORC1 [30, 31]. Both wild type and TSC2-deficient MEFS were also deficient for p53 since TSC2 deficiency results in cell senescence that is rescued by p53 deletion [32]. Similar to wild-type eIF2α S/S MEFs, PEITC also inhibited global mRNA translation in these MEFs (Figure 3D). However, there was no evidence that inhibition of metabolic labeling by PEITC differed between wild-type and TSC2-deficient cells. Thus, consistent with results obtained using rapamycin in MCF7 cells (Figure 1D), PEITC-mediated inhibition of mTORC1 activity does not appear to make a major contribution to effects of PEITC on global mRNA translation. However, it is important to recognise that, despite the clear importance of eIF2α phosphorylation for inhibition of mRNA translation in MEFs revealed by analysis of eIF2α (A/A) mutant cells, higher concentrations of PEITC did inhibit mRNA translation in the absence of eIF2α phosphorylation (Figure 3B). These effects may be mediated via secondary inhibitory effects on mTORC1 and/or additional pathways.

We attempted to identify the kinase(s) responsible for PEITC-induced eIF2α phosphorylation focusing on the ER-resident eIF2α Ser51-specific kinase PERK. PEITC increased PERK phosphorylation at Thr980 (a marker of activation) in both eIF2α S/S and A/A MEFs (data not shown). However, siRNA mediated knock-down of PERK in MCF7 cells (~75% reduction of PERK expression) only partly decreased PEITC-induced eIF2α phosphorylation by ~50% (data not shown). Thus, the kinase(s) required for PEITC-induced eIF2α phosphorylation remains to be identified. Despite optimization of transfections, it is not clear whether there was sufficient residual PERK to mediate effective eIF2α phosphorylation, or whether response involves multiple, perhaps compensating, kinases.

### PEITC inhibits mRNA translation and BCR signaling in CLL cells

We next probed effects of PEITC in primary CLL cells. We investigated effects of PEITC on both basal (ie, in unstimulated cells treated with a control antibody) and anti-IgM-induced mRNA translation. mRNA translation was quantified at 24 hours, consistent with our previous study [24]. Anti-IgM signaling responses vary between samples and we selected a cohort of samples all of which were considered as signaling responsive based on our previous criteria [33]. The features of the selected samples (some of which were analysed in our previous study), including sIgM expression and signal capacity, and prognostic markers (*IGHV* mutation status, ZAP70/CD38 expression) are shown in Supplementary Table S1.

**Figure 4:**
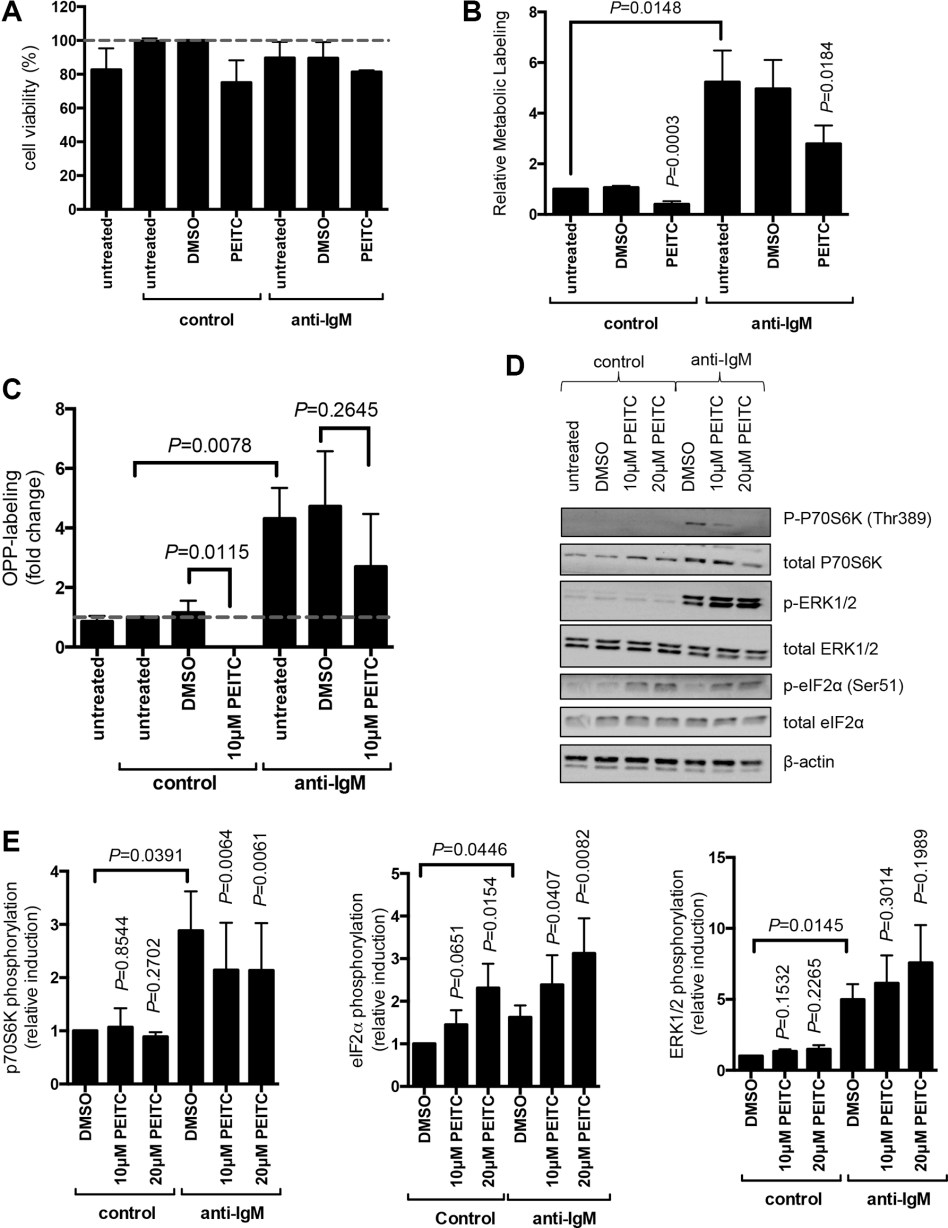
Effect of PEITC on basal and anti-IgM-induced mRNA translation in primary human leukemia cells (**A**–**C**) CLL samples (*n* = 7) were treated with Q-VD-OPh (5 μM) and then stimulated with anti-IgM or control antibody. After 17 hours, cells were treated with PEITC (10 μM) or DMSO as a solvent control for an additional five hours. (A) Cell viability was determined by Annexin V staining. The graph shows the mean (± SEM) percentage of viable (annexin V^−^) cells relative to control antibody/DMSO-treated cells derived from three independent experiments, each performed in duplicate. (B) mRNA translation was quantified using metabolic labeling, or (C) OPP-labelling. (**D**, **E**) CLL samples were treated with PEITC (10 or 20 μM) or DMSO as a control and stimulated with anti-IgM, or control antibody for 60 minutes, expression of phosphorylated and total p70S6K, ERK1/2 and eIF2α was analyzed by immunoblotting. (D) Representative immunoblot. (E) Quantitation of multiple samples following 60 minutes of treatment (*n* = 5–8 for p70S6K; *n* = 3–6 for ERK1/2; *n* = 4 for eIF2α). Graphs show means (± SEM) with values for control cells set to 1.0. Statistical comparisons between groups are shown (Student's *t*-test).

We first investigated effects of PEITC using metabolic labeling. CLL cells undergo spontaneous apoptosis *in vitro*, and this can be accelerated by PEITC [34]. We selected a maximum concentration of 10 μM for our studies, and restricted exposure to PEITC to the final 5 hours of the 24 hour anti-IgM treatment to minimise potentially confounding effects of apoptosis. Cells were also treated with the caspase inhibitor Q-VD-OPh to suppress apoptosis; cell viability analysis confirmed that there was no evidence for significant PEITC-induced cell killing under our experimental conditions (Figure 4A). Consistent with our recent findings, [24] anti-IgM increased metabolic labeling in CLL cells. PEITC significantly inhibited basal and anti-IgM-induced metabolic labeling (both by~50%) (Figure 4B).

Although the samples selected for study typically contained a high proportion of CLL B-cells, we also used OPP-labeling [35] to specifically quantify mRNA translation within the malignant clone. OPP-labeling was combined with staining with anti-CD19 and anti-CD5 antibodies to identify CLL cells, and scatter analysis was used to gate on viable cells [24]. OPP-labeling confirmed that PEITC inhibited both basal and anti-IgM-induced mRNA translation within the malignant clone (Figure 4C). In this assay, PEITC completely blocked basal translation. Effects on anti-IgM-induced mRNA translation were partial (~30% reduction) and did not achieve statistical significance.

We investigated effects of PEITC on phosphorylation of eIF2α and p70S6K at 60 minutes post-stimulation. PEITC was tested at both 10 and 20 μM in these shorter term experiments. As shown previously, [24, 36] anti-IgM alone increased p70S6K phosphorylation in CLL cells (by ~3-fold), but had only modest effects on eIF2α phosphorylation (~50% increase; Figure 4D, 4E). PEITC had no effect on p70S6K phosphorylation in control cells, but significantly inhibited the response to anti-IgM (~40% reduction). By contrast, PEITC significantly increased eIF2α phosphorylation in the presence or absence of anti-IgM (Figure 4D, 4E). Therefore, PEITC counters anti-IgM-induced mTORC1 activation, and activates eIF2α phosphorylation in the presence or absence of anti-IgM in CLL cells. Due to very small amount of cytoplasm in CLL cells, we were unable to investigate the formation of stress granules in these cells.

As a control, we also investigated effects of PEITC on phosphorylation of ERK1/2 which is activated downstream of the BCR independent of mTORC1 and eIF2α. PEITC had no effect on ERK1/2 phosphorylation in control cells, and did not inhibit anti-IgM-induced ERK1/2 phosphorylation (Figure 4D, 4E). In fact, PEITC modestly increased anti-IgM-induced ERK1/2 phosphorylation, consistent with effects of PEITC in other cell types [37].

### PEITC inhibits anti-IgM-induced transcription and translation of the oncogene MYC

**Figure 5:**
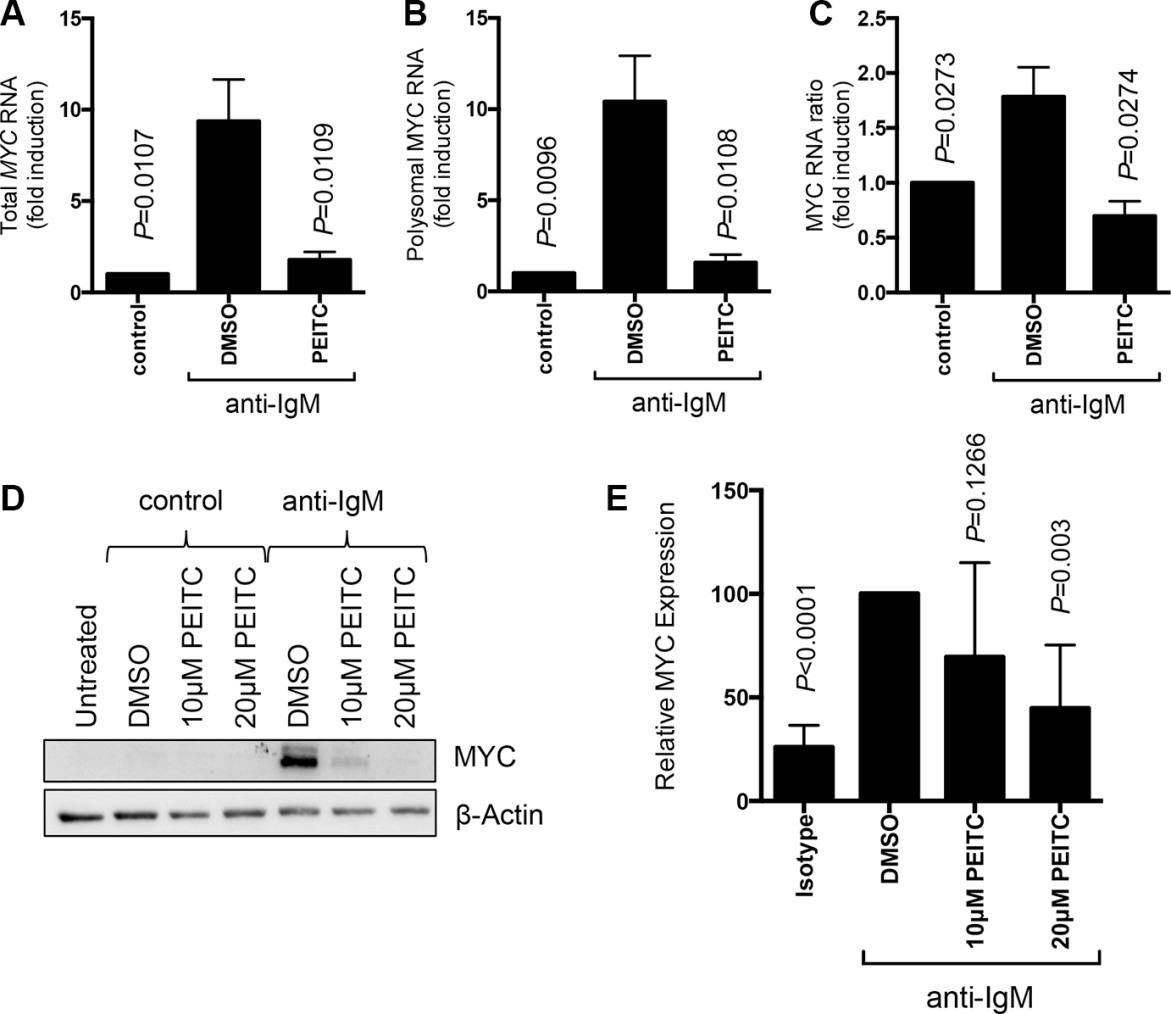
Effect of PEITC on MYC regulation CLL cells (*n* = 7) were stimulated with anti-IgM or control antibody. After 17 hours, cells were treated with PEITC (10 μM) or DMSO as a solvent control for an additional 5 hours. (**A**–**C**) Analysis of monosome/polysome associated *MYC* mRNA using Q-PCR. Graphs show quantitation of (A) total *MYC* mRNA (monosomal plus polysomal); (B) polysome-associated *MYC* mRNA; and (C) polysome/monosome ratio for *MYC* mRNA. Graphs show means (± SEM) with values with control antibody treated cells set to 1.0. Statistical significance of differences between indicated groups are shown (Student's *t*-test). (**D**) Analysis of MYC protein expression by immunoblotting and (**E**) quantification.

To further investigate effects of PEITC on mRNA translation in primary CLL cells, we examined effects of PEITC on MYC. We used Q-PCR to quantify *MYC* mRNA in fractions derived from polysome profiles. Anti-IgM enhanced both *MYC* RNA transcription and translation since anti-IgM increased the total amount of *MYC* mRNA recovered from the fractions, as well as the amount of *MYC* mRNA specifically present in polysome associated fractions (Figure 5A–5C) [24]. Translation responses were observed regardless of whether we determined the absolute amount of *MYC* mRNA in polysome fractions (Figure 5B) or the proportion of polysome-associated *MYC* mRNA (Figure 5C) which measures translation changes, independent of changes in the overall levels of *MYC* mRNA. PEITC significantly reduced anti-IgM-induced *MYC* mRNA transcription and translation (Figure 5A–5C). PEITC also effectively decreased anti-IgM-induced MYC expression (Figure 5D, 5E).

### PEITC enhances inhibitory effects of ibrutinib on mRNA translation and viability in CLL cells

**Figure 6:**
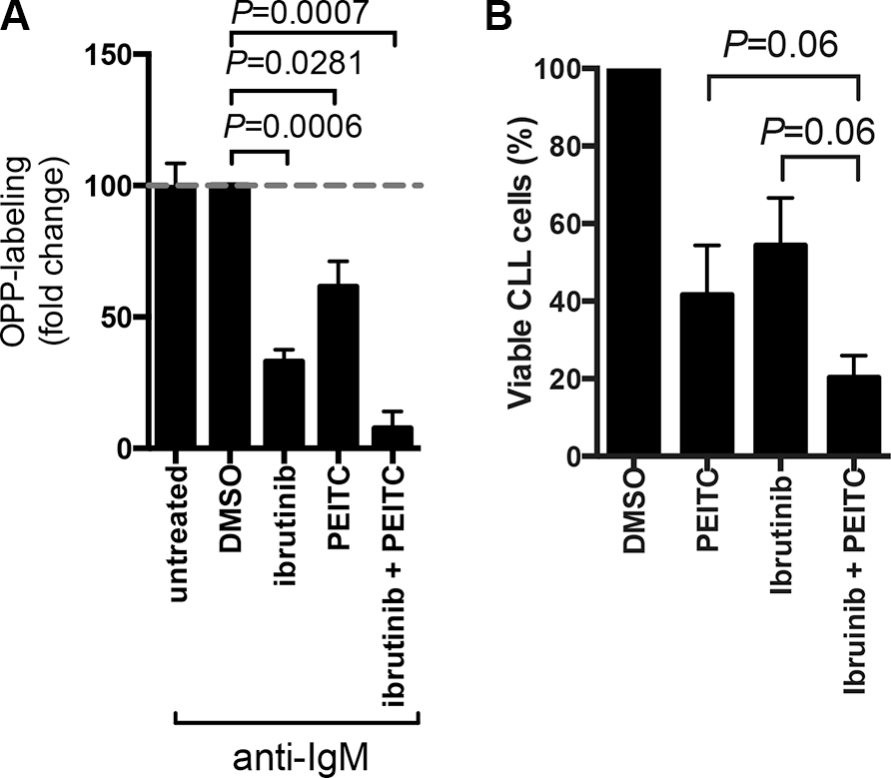
Effect of PEITC in combination with ibrutinib on cell viability and mRNA translation (**A**) CLL cells were stimulated with anti-IgM for a total of 24 hours in the presence or absence of ibrutinib (10 μM) and/or PEITC (10 μM). For ibrutinib treatment, cells were pretreated with drug for 1 hour before addition of anti-IgM. For PEITC, cells were treated with drug for the final 5 hours of BCR stimulation. DMSO was used as a solvent control. mRNA translation was then quantified by OPP-labeling. Graph show means (± SEM) with values for anti-IgM treated cells set to 100%. Statistical significance of differences between indicated groups are shown (Student's *t*-test), *n* = 4. (**B**) CLL cells were treated with 10 μM ibrutinib for 24 hours alone, 10 μM PEITC alone for 5 hours, a combination of 10 μM ibruintib for 24 hours and 10 μM PEITC for the final 5 hours. DMSO was used a solvent control. Cell viability was determined by AnnexinV PI staining. Bars (± SEM) represent the percentage of viable cells (annexinV and PI double negative cells), *n* = 5. *P* values are indicated from paired Wilcoxon non-parametric *t*-test.

We previously demonstrated that the BTK inhibitor, ibrutinib, partially inhibited anti-IgM-induced mRNA translation [38]. To investigate whether PEITC could enhance responses to ibrutinib in CLL, we analysed effects of combinational treatment on anti-IgM-induced OPP-labeling. The combination of PEITC and ibrutinib caused significant greater inhibition of anti-IgM-induced mRNA translation compared to either agent alone (Figure 6A). Moreover, the combination of PEITC and ibrutinib also induced higher levels of CLL cell death compared to single agent treatments (Figure 6B).

## DISCUSSION

Dysregulation of protein synthesis plays a critical role in carcinogenesis and there is considerable interest in chemical compounds that inhibit mRNA translation as potential anti-cancer agents [17]. We previously showed that the phytochemical PEITC inhibited mTORC1 activity and downstream *HIF1A* mRNA translation [4]. In this work, we investigated effects of PEITC on mRNA translation using both established cell lines and primary leukemic cells.

We demonstrated that PEITC rapidly inhibited global mRNA translation and used genetically-manipulated MEFs to define the role of mTORC1 inhibition and eIF2α phosphorylation in this response. Although mTORC1 inhibition seems to mediate PEITC-induced inhibition of *HIF1A* mRNA translation (which is highly dependent on mTORC1 signalling), [19, 20] mTORC1 inhibition appeared to make little contribution to effects of PEITC on global mRNA translation. Thus, mutation of eIF2α to prevent its activation via phosphorylation significantly reduced cell sensitivity to PEITC-mediated translational inhibition whereas deletion of TSC2 (which prevents PEITC-mediated mTORC1 inhibition) [4] did not alter responses. However, it is important to note that PEITC retained some inhibitory activity in eIF2α mutant MEFs. It is possible that mTORC1 inhibition contributes to PEITC-mediated translational inhibition alongside eIF2α phosphorylation, but does not play a substantial role alone. However, we do not exclude the possibility that PEITC may exert effects on additional regulatory pathways not investigated in these experiments.

The mechanisms by which PEITC induces eIF2α phosphorylation remain to be determined. Similar to a recent study in ovarian cancer cell lines, [39] we demonstrated that PEITC activated the ER-resident eIF2α kinase PERK in MCF7 cells. PEITC leads to the accumulation of reactive oxygen species (ROS) via depletion of glutathione, including in MCF7 [40] and CLL cells [34]. The ER is normally a relatively oxidizing environment and may therefore be particularly susceptible to PEITC-induced increases in ROS. However, results using PERK-specific RNAi were inconclusive, possibly due to residual PERK expression despite optimization of knock-down conditions.

Long-term culture of established cell lines is likely to be associated with selection of variants with high mRNA translation and a major goal of our study was to extend analysis to leukemic primary cells, using CLL as a model. PEITC inhibited both basal and anti-IgM-induced global mRNA translation in CLL cells. Overall, PEITC appeared more effective in inhibiting basal, compared to anti-IgM-induced global mRNA translation. Pathway analysis revealed that PEITC increased eIF2α phosphorylation in the presence or absence of sIgM stimulation, and partially decreased anti-IgM-induced p70S6K phosphorylation. Thus, in the absence of sIgM stimulation, where mRNA translation is low and mTORC1 is not activated, inhibitory effects of PEITC appear to be mediated via eIF2α phosphorylation. Following stimulation, mRNA translation inhibition may be mediated by combined effects of PEITC on eIF2α and mTORC1. The partial inhibition of anti-IgM-induced p70S6K phosphorylation by PEITC may explain why PEITC only partially inhibited induced global mRNA translation. Alternately, it is possible that sIgM stimulation activates additional pathways not studied here to promote mRNA translation and that these operate independently of PEITC. Potential candidates include the MNK1/2 kinases activated downstream of ERK/p38 MAPK which can also enhance mRNA translation in other cell systems [7]. Interestingly, in contrast to partial effects on global protein synthesis, PEITC completely suppressed anti-IgM-induced *MYC* RNA translation. *MYC* RNA has a highly structured 5′-UTR and its translation therefore may be particularly affected by modest reductions in mTORC1-signalling.

Despite substantial improvements in patient outcomes following the introduction of new targeted agents such as ibrutinib, CLL remains an incurable disease with a pressing need for novel therapeutic approaches. In our previous study, [38] we demonstrated that ibrutinib only partially inhibited anti-IgM-induced mRNA translation. Importantly, here we demonstrate that the combination of PEITC and ibrutinib resulted in almost complete blockage of anti-IgM-induced mRNA translation and that combined drug treatment was also associated with enhanced cytotoxic activity. Pronounced inhibitory effects on mRNA translation may stem from combined effects on both the mTORC1 and eIF2α-mediated arms of translational control. PEITC has been shown previously to promote apoptosis of CLL cells [34] and our study provides a potential rationale for combinatorial testing using PEITC to enhance responses to ibrutinib.

In conclusion, PEITC exerts inhibitory effects on both global and oncogene-specific mRNA translation, including MYC, via multiple pathways. These effects may contribute to both the chemo-preventive and anti-cancer effects of PEITC, and could be used to boost the efficacy of other agents, such as ibrutinib.

## MATERIALS AND METHODS

### Cell culture

The MCF7 cell line was obtained from American Type Culture Collection and cultured in complete Dulbecco's Modified Eagle Medium (DMEM), ie supplemented with 10% (v/v) bovine fetal serum, 2 mM L-glutamine and 1% (v/v) penicillin/streptomycin mix (all PAA Somerset, UK). MEFs containing wild type eIF2α (MEF eIF2α S/S) and MEFs containing the homozygous eIF2α Ser51Ala mutation (MEF eIF2α A/A) were kindly provided by Professor R. Kaufman, University of Michigan Medical Center, USA [29]. TSC2-deficient (p53^−/−^TSC2^−/−^) and matched control MEFs (p53^−/−^TSC2^+/+^) were kindly provided by Drs. A Tee (Cardiff University, UK) and D. Kwiatkowski (Brigham and Women's Hospital, Boston, MA, USA) [32]. MEFs were cultured in complete DMEM.

Primary malignant B cells were obtained from the blood of CLL patients, as described [24]. Patients provided written informed consent in accordance with Ethics Committee approvals and the Declaration of Helsinki. Briefly, heparinized peripheral blood mononuclear cells were isolated using Lymphoprep (Axis-Shield, Oslo, Norway) and cryopreserved. Following recovery, cells were rested for one hour at 37°C prior to use. Cell viability determined by trypan blue exclusion was ≥ 90% and the median proportion of CD5^+^CD19^+^ CLL cells was 95% (range 82–99%). Molecular markers and sIgM signaling capacity were analyzed as previously described [33].

PEITC and the caspase inhibitor Q-VD-OPh were from Sigma Chemicals (Poole, UK). Cycloheximide (Sigma Chemicals) was used as a positive control for inhibition of RNA translation and was used at 10 μg/ml in the final five minutes of incubation. For B-cell receptor (BCR) stimulation, CLL samples were incubated with bead-bound goat F(ab')_2_ anti-human IgM or control antibodies, as described [41]. Ibrutinib was from SelleckChem (Suffolk, UK).

### mRNA translation assays

Polysome profiling, ^35^S-Met/Cys metabolic labeling and OPP assays [35] were performed as previously described [24]. For MCF7 cells and MEFs, metabolic labeling was performed for 2 hours and polysome profiling used 3 × 10^6^ cells. For CLL cells, metabolic labeling was performed during the final 5 hours of cell incubations and polysome profiling used 10 × 10^7^ cells.

### Immunoblot analysis

Immunoblot analysis was performed using the following antibodies; anti–T^202^/Y^204^-phosphorylated ERK1/2, anti-ERK1/2, anti-T^389^-phosphorylated p70S6K, anti-p70S6K, anti-S^51^-phosphorylated eIF2α, anti-eIF2α (all Cell Signaling, Herts, UK), anti-MYC (9E10; Calbiochem, Nottingham, UK) anti-β-actin (Sigma Chemicals). Analysis used equal protein loading following quantitation of protein content using the BioRad Protein Assay (BioRad, Hemel Hempstead, UK). Secondary antibodies were HRP-conjugated rabbit, mouse or goat antibodies (Dako, Cambridgeshire, UK) and images were captured using the ChemiDoc-It Imaging System with a BioChemi HR camera (UVP, Cambridge, UK). Immunoblot signals were quantified using ImageJ (http://imagej.nih.gov/ij/). For quantitation of phosphorylation, expression values were normalized to the loading control (β-actin) and then the relevant total protein. For quantitation of MYC, expression values were normalized to β-actin.

### Immunofluorescence and confocal microscopy

Immunofluorescence and confocal microscopy was carried out on cell lines grown on coverslips and fixed using 4% (w/v) paraformaldehyde (BDH Laboratory Supplies, Poole, UK) and permeabilised using 0.1% (v/v) Triton X-100 (Sigma Chemicals). Staining was carried out using anti-eIF3b (Santa Cruz, Heidelberg, Germany), anti-TIA-1 (Abcam, Cambridge, UK) and DAPI (Sigma Chemicals) was used as a nuclear stain. Coverslips were mounted onto microscopy slides using mounting media (Dako). Cells were imaged on the Olympus immunofluorescence microscope and captures using the Olympus 1 × 81 Camera (Olympus, Essex, UK). For confocal microscopy images were captures on a Leica microscope using the LAS AF software (Leica Microsystems, Milton Keynes, UK).

### Cell viability assays

Cell viability was analyzed using annexin V/propidium iodide staining as described [28].

### Statistics

Statistical comparisons were performed using Student's *t*-tests (Prism 6 software, GraphPad Software, La Jolla, CA, USA).

## SUPPLEMENTARY MATERIALS FIGURES AND TABLES


