# Supplementary material for: PEITC-mediated inhibition of mRNA translation is associated with both inhibition of mTORC1 and increased eIF2α phosphorylation in established cell lines and primary human leukemia cells
Source: Oncotarget. 2016 Aug 27;7(46):74807–19. doi: 10.18632/oncotarget.11655 (PMC5342703; doi:10.18632/oncotarget.11655)
Supplement: Supplementary file 1 [file oncotarget-07-74807-s001.pdf]

PEITC-mediated inhibition of mRNA translation is associated with both inhibition of mTORC1 and increased eIF2α phosphorylation in established cell lines and primary human leukemia cells

Supplementary Materials

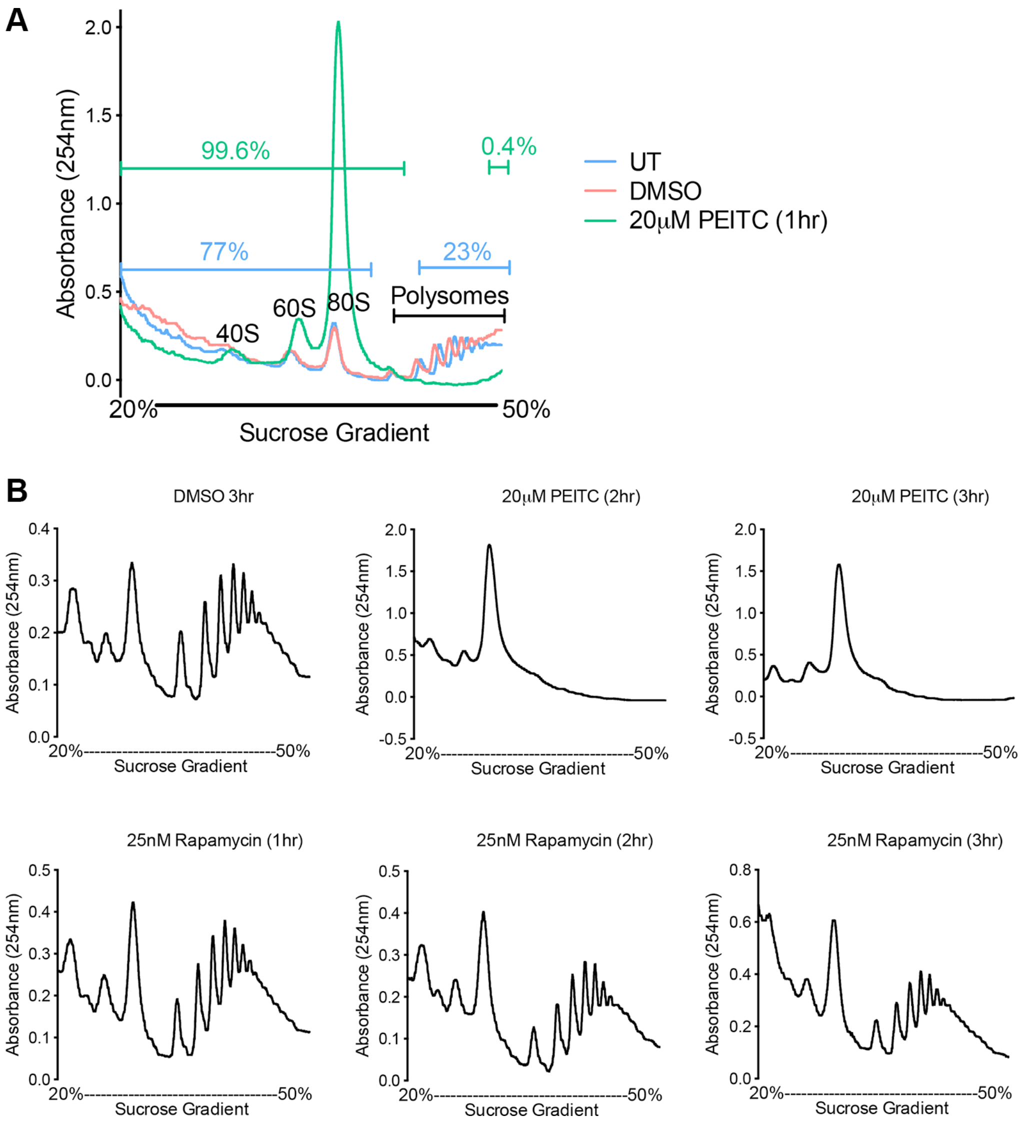

Supplementary Figure S1: Polysome Profiles of MCF7 Cells Following Treatment with PEITC. MCF7 cells were untreated, treated with 20 μM PEITC or equivalent DMSO for one hour prior to polysome profiling. (**A**) Complete polysome profiles with the 40S, 60S, 80S and polysome peaks identified. Percentages represent the area under the curve (prism). (**B**) MCF7 cells were treated with 20 μM PEITC, 25 nM Rapamycin or equivalent DMSO for one, two or three hours prior to polysome profiling. Data presented are representative of three independent repeats.

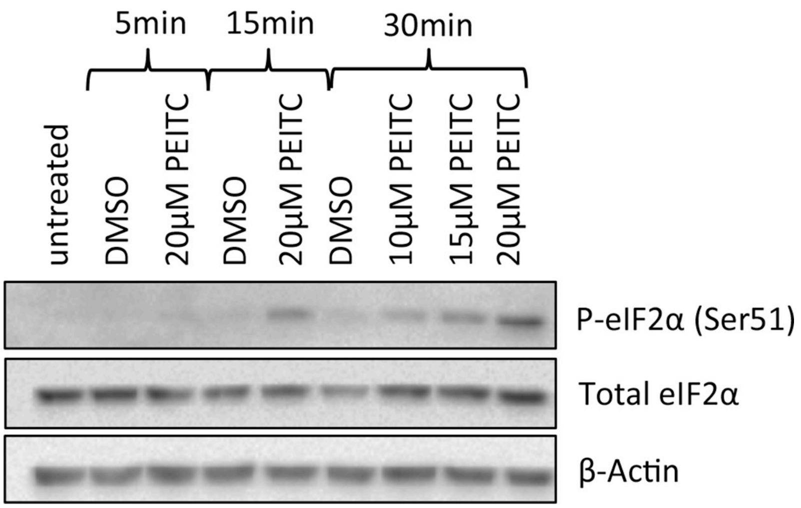

Supplementary Figure S2: Effect of PEITC on eIF2α phosphorylation. MCF7 cells were treated with the indicated concentrations of PEITC or DMSO for up to 30 minutes. Untreated cells were analyzed at the start of the experiment. Total and phosphorylated eIF2α, and β-actin, were analyzed by immunoblotting. Representative results from three independent experiments.

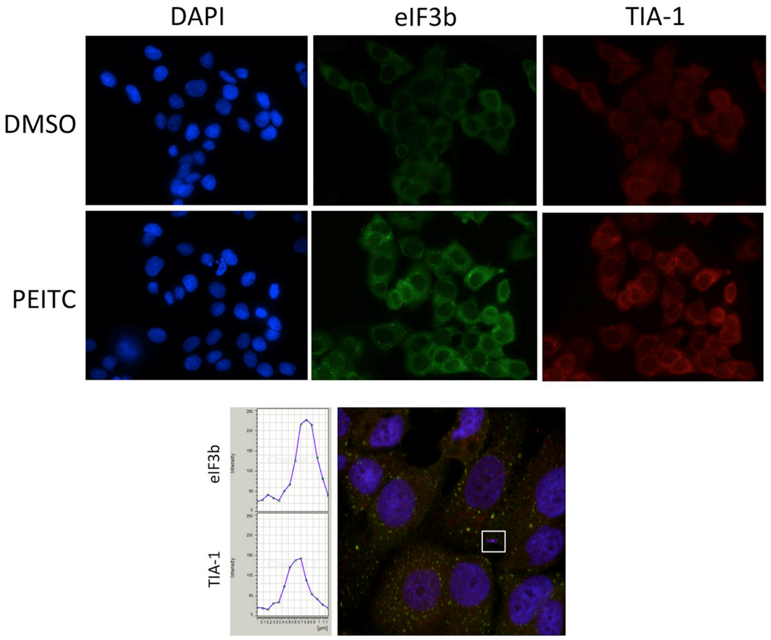

Supplementary Figure S3: Formation of stress granules in MCF7 cells following treatment with PEITC – color images. See Figure 2 of main manuscript for full description.

Supplementary Table S1: Details of samples used for *in vitro* studies

Sample[a]	*IGHV* gene	*IGHV* status[b]	Stage[c]	Therapy[d]	CLL (% cells)[e]	ZAP-70 (% cells)	CD38 (% cells)	sIgM (MFI)	sIgM signal (% cells)[f]
279	V3-30	M	II	N	97	0	0	23	12
348A	V3-15	M	0	N	93	0	4	66	19
483	V2-5	M	II	N	90	3	0	49	5
494	V3-15	M	I	N	95	19	1	29	6
505	V1-69	U	I	N	94	14	14	45	17
508A	V2-26	M	II	N	93	13	13	46	19
513	V1-69	U	I	N	98	2	1	80	62
523A	V3-72	M	0	N	89	17	0	50	61
526	V3-30	U	II	N	96	40	3	27	7
531A	V4-39	U	I	Y	97	17	0	40	41
558	M4-31	M	II	N	95	4	0	16	39
561	V3-48	M	I	N	95	24	2	39	50
566	V3-11	U	I	N	95	57	9	70	7
583	V1-69	U	I	N	91	39	1	56	16
595A	V3-7	M	I	N	95	3	74	26	10
604A	V3-30	M	II	N	92	2	0	94	42
632	nd	nd	I	N	96	90	71	46	69
633	V3-7	M	I	N	82	0	0	45	58
635	V3-21	U	I	N	87	12	1	67	82
643	V6-1	M	I	N	91	0	0	25	25
684A	V3-15	M	0	N	90	8	8	31	14
695	V3-21	U	I	N	98	1	7	80	82
704	V4-39	M	IV	Y	99	1	41	31	70

[a]Where suffix is not shown, this is the first sample obtained from that patient, typically obtained shortly after diagnosis. A indicate subsequent samples. [b]*IGHV* mutation status. M, mutated; U, unmutated. [c]clinical stage of disease according to Rai classification at time of sample collection. [d]Indicates whether therapy for CLL was given prior to sample collection. [e]Percentage of $CD5^{+}CD19^{+}$ cells. [f]Maximal percentage of cells with increased intracellular calcium following treatment with soluble anti-IgM. nd, not determined.
